# Assessing Cognitive Abilities in High-Performing Cochlear Implant Users

**DOI:** 10.3389/fnins.2018.01056

**Published:** 2019-01-15

**Authors:** Jake Hillyer, Elizabeth Elkins, Chantel Hazlewood, Stacey D. Watson, Julie G. Arenberg, Alexandra Parbery-Clark

**Affiliations:** ^1^School of Medicine, Oregon Health & Science University, Portland, OR, United States; ^2^Auditory Research Laboratory, Center for Hearing and Skull Base Surgery, Swedish Neuroscience Institute, Seattle, WA, Unites States; ^3^Massachusetts Eye and Ear Infirmary, Department of Otolaryngology, Harvard Medical School, Boston, MA, United States

**Keywords:** cochlear implant, cognitive skills, speech perception, clinical outcome, working memory

## Abstract

Despite being considered one of the most successful neural prostheses, cochlear implants (CIs) provide recipients with a wide range of speech perception performance. While some CI users can understand speech in the absence of visual cues, other recipients exhibit more limited speech perception. Cognitive skills have been documented as a contributor to complex auditory processing, such as language understanding; however, there are no normative data for existing standardized clinical tests assessing cognitive abilities in CI users. Here, we assess the impact of modality of presentation (i.e., auditory-visual versus visual) for the administration of working memory tests in high-performing CI users in addition to measuring processing speed, cognitive efficiency and intelligence quotient (IQ). Second, we relate performance on these cognitive measures to clinical CI speech perception outcomes.

**Methods:** Twenty one post-lingually deafened, high-performing, adult CI users [age range: 52–88 years; 3 unilateral CI, 13 bimodal (i.e., CI with contralateral hearing aid), 5 bilateral CI] with clinical speech perception scores (i.e., AzBio sentences in quiet for the first-ear CI) of ≥60% were recruited. A cognitive test battery assessing auditory-visual working memory (AVWM), visual working memory (VWM), processing speed, cognitive efficiency and IQ was administered, in addition to clinical measures of speech perception in quiet (i.e., AzBio sentences in quiet). AzBio sentences were assessed in two conditions: first-ear CI only, and best-aided everyday wearing condition. Subjects also provided self-reported measures of performance and benefit from their CI using standardized materials, including the Glasgow Benefit Inventory (GBI) and the Nijmegen Cochlear Implant questionnaire (NCIQ).

**Results:** High-performing CI users demonstrated greater VWM than AVWM recall. VWM was positively related to AzBio scores when measured in the first-ear CI only. AVWM, processing speed, cognitive efficiency, and IQ did not relate to either measure of speech perception (i.e., first-ear CI or best-aided conditions). Subjects’ self-reported benefit as measured by the GBI predicted best-aided CI speech perception performance.

**Conclusion:** In high-performing CI recipients, visual presentation of working memory tests may improve our assessment of cognitive function.

## Introduction

Cochlear implants (CIs) provide an effective treatment option for individuals with severe to profound hearing loss who no longer benefit from other assistive devices such as hearing aids (Dowell, 2012). However, the ability to predict post-implantation hearing performance (e.g., speech perception) is challenging (Holden et al., 2013; van Eijl et al., 2016). Among several factors investigated, age at implantation and duration of hearing loss have shown to be negatively related to speech perception, and have been estimated to predict between 10 and 22% of the variance in speech perception scores (Blamey et al., 1996; Lazard et al., 2012b; Holden et al., 2013). Other consistent predictors of speech perception are surgical factors (e.g., electrode number and placement; Holden et al., 2013), however, these tend to account for less variance than age at implantation and duration of hearing loss, meaning that a large portion remains unexplained. Early work highlighted intelligence quotient (IQ; Punch et al., 1987), the ability to use non-verbal communication strategies (Gantz et al., 1993), reading span measures (Lyxell et al., 1998) and verbal learning ability (Heydebrand et al., 2007) as factors related to adult CI performance, although others have documented no such relationships (van Dijk et al., 1999; Collison et al., 2004; Holden et al., 2013). While some evidence suggests an auditory-cognitive link between working memory (WM) ability and speech perception in normal hearing (NH) middle-aged and older adults, as well as in hearing impaired (HI) individuals, especially in noise (van Rooij and Plomp, 1990; Akeroyd, 2008; Houtgast and Festen, 2008; Zekveld et al., 2011; Humes et al., 2013; Füllgrabe and Rosen, 2016), contributions of central cognitive factors (i.e., processes such as attention, memory, and problem solving used to complete complex tasks) to speech performance in adult cochlear implant recipients have only recently been explored (Moberly et al., 2017a; Pisoni et al., 2018).

For successful speech perception, post-lingually deafened adult CI users relate speech signals to long-term memory representations of lexical and phonological knowledge (Pisoni and Cleary, 2003). From a “bottom–up” perspective, CIs provide a spectrally and temporally degraded signal which increases the level of ambiguity for speech intelligibility. One way to navigate degraded sensory information is for a listener to apply “top–down” processes such as linguistic knowledge related to semantics, syntax, and phonological structure (Ahissar et al., 2008). Furthermore, evidence suggests that HI individuals resort to other top–down processes such as signal deduction or phonemic restoration to improve speech understanding (Başkent, 2012). Top–down signal deduction, however, increases listening effort and reduces available cognitive resources (Pichora-Fuller, 2006b). Consequently, CI users, whose access to speech is via a spectrally degraded signal, may rely more heavily on cognitive functions.

Meta-analyses that have examined the relationship between cognition and speech perception in older HI individuals suggest that while audibility is the primary factor in predicting speech perception, cognitive abilities also contribute to variance in performance (Akeroyd, 2008; Houtgast and Festen, 2008). Furthermore, studies that have ensured audibility of test materials via utilization of hearing aids or modification of those test materials (i.e., auditory versus visual presentation, adjustment of auditory presentation levels or acoustic filtering) have also revealed relationships between cognitive ability and speech perception (Humes et al., 2013; Smith and Pichora-Fuller, 2015). In order to examine the contributions of cognitive mechanisms to language processing, it is critical to obtain an accurate measurement of cognitive skills (Arlinger et al., 2009). Many commonly used cognitive tests are presented in an auditory-only modality (e.g., Forward or Reverse Digit Span, Listening Span), yet use of this presentation method to assess cognitive function in individuals with hearing loss, without accounting for audibility, may impact the quantification of their cognitive ability (Dupuis et al., 2015). As such, incorporating visually-based test materials when assessing cognitive skills in CI users may avoid the potential pitfalls associated with auditory-only based tests (Pisoni et al., 2018), and may in turn improve the specificity of cognitive assessments in HI individuals (Weinstein and Amsel, 1986).

It is unclear how modality of presentation of cognitive measures impacts performance in adult CI users. WM is the temporary storage and processing mechanism whereby encoded information can be further analyzed and manipulated. WM task performance is of particular interest in CI users because WM is associated with speech perception abilities of pediatric CI users (Pisoni and Geers, 2000; Dawson et al., 2002; Pisoni and Cleary, 2003), but the role of WM in adult CI user speech perception ability is not well-understood (Moberly et al., 2018c; Pisoni et al., 2018). The current study therefore examined WM via the visual-only and auditory-visual modalities in high-performing CI users. High-performing CI recipients, defined as patients with clinical speech perception scores (i.e., AzBio sentences in quiet) of ≥60% were enrolled to maximize comprehension of test instructions and intelligibility of auditorily-presented cognitive test items.

Current research is undecided on whether processing speed, defined as the rate at which information is treated or an operation is performed, contributes to speech perception in CI users (Hua et al., 2017; Purdy et al., 2017; Moberly et al., 2018a). Furthermore, because WM allows for auditory stimuli to be processed, manipulated, and partially stored, processing speed and WM are intricately related when it comes to speech perception (Pichora-Fuller, 2006a). Processing speed of auditory information may be impacted by the complexity and speed of auditory message(s), which in turn affect WM capacity (i.e., manipulation and storage; Stewart and Wingfield, 2009). Despite processing speed and WM being related, they have been studied as separate constructs in CI research. As such, measures of processing speed and cognitive efficiency (i.e., a combination of processing speed and short-term WM; Vernon, 1983; McGrew et al., 2014) were also assessed. The second goal of the present study was to define the relationship between cognitive measures [i.e., auditory-visual working memory (AVWM), visual working memory (VWM), processing speed, and cognitive efficiency] and speech perception ability in high-performing CI users. We hypothesized that CI users would benefit from the visual presentation of test items and demonstrate higher levels of recall for visually over auditorily-presented items. We also anticipated that if visually-presented cognitive tests provided a more accurate quantification of underlying cognitive skills in CI users, visually-presented cognitive tests would relate to a greater degree with clinical metrics of speech perception in quiet.

## Materials and Methods

Twenty one experienced CI users (>6 months listening experience, mean = 34.38 months, *SD* = 22.03 months, range of 10–79 months), between the ages of 52 and 88 years were recruited from the patient pool at the Center for Hearing and Skull-Based Surgery at The Swedish Neuroscience Institute in Seattle, WA, United States (see Table 1 for participant details). Inclusion criteria required patients to have clinical speech perception scores in quiet at or above 60% with their first-ear CI [i.e., AzBio sentences presented at 60 decibel (dB) sound pressure level (SPL) delivered via a loudspeaker (GN Otometrics Astera Sound Field Speakers) at 0 degrees azimuth], no recorded symptoms or diagnosis of dementia, no report of cognitive decline and no congenital etiology or pre-lingual hearing loss. All participants were native English speakers, had at least a high school education and demonstrated normal IQ scores, as measured by the Test of Non-verbal Intelligence – 4^th^ Edition (TONI-4; Brown et al., 2010). Three participants were unilateral CI users, 5 were bilateral CI users, and 13 were bimodal users with a CI and a contralateral hearing aid. In terms of implant manufacturer, 1 had Advanced Bionics, 15 had Cochlear Americas, and 5 had Med-El. Experienced CI users (>6 months listening experience) were recruited because maximum comfortable (C) level and threshold (T) level are optimally achieved after 6 months of use (Gajadeera et al., 2017). All participants who regularly wore glasses were allowed to keep them on for the duration of the study session; no participants disclosed visual impairments. All testing procedures were approved by the Swedish Medical Center Institutional Review Board (#SWD565S-14); all participants provided informed written consent.

**Table 1:** Participant demographics.

CI status	CI device	Etiology of hearing loss	Use of 1^st^ CI (Mo.)	Use of 2^nd^ CI (Mo.)
Unilateral	Cochlear Americas	Potentially genetic	17	–
Unilateral	Cochlear Americas	Potentially genetic	15	–
Unilateral	Cochlear Americas	Sudden hearing loss	15	–
Bimodal	Advanced Bionics	Potentially genetic	14	–
Bimodal	Cochlear Americas	Potentially genetic	15	–
Bimodal	Cochlear Americas	Meniere’s disease	15	–
Bimodal	Cochlear Americas	Potentially genetic	12	–
Bimodal	Cochlear Americas	Noise exposure, potentially genetic	66	–
Bimodal	Cochlear Americas	Unknown	52	–
Bimodal	Cochlear Americas	Meniere’s disease, family history, noise exposure	16	–
Bimodal	Cochlear Americas	Progressive, unknown	10	–
Bimodal	Cochlear Americas	Noise exposure, potentially genetic	12	–
Bimodal	Med-El	Noise exposure	37	–
Bimodal	Med-El	Unknown	61	–
Bimodal	Med-El	Unknown	32	–
Bimodal	Med-El	Unknown	62	–
Bilateral	Cochlear Americas	Rheumatic fever, chronic otitis media	51	32
Bilateral	Cochlear Americas	Noise exposure	79	49
Bilateral	Cochlear Americas	Potentially genetic	46	13
Bilateral	Cochlear Americas	Noise exposure	50	30
Bilateral	Med-El	Sudden hearing loss	45	41


### Speech Testing

#### AzBio Sentences Test

The AzBio Sentences Test (Spahr et al., 2012) was administered to participants to assess their speech perception abilities. The test was administered in a sound-proof booth using recordings of 20 sentences spoken by two male and two female talkers. Sentences ranged from 4 to 12 words, all of which were keywords for scoring purposes, and were spoken by one talker at a time in a conversational style with minimal contextual cues (e.g., “She missed a week of work and nobody noticed”). The sentences were presented at 60 dB SPL from a loudspeaker (GN Otometrics Astera Sound Field Speakers) at 0 degrees azimuth, 2 m from the participant, who was instructed to repeat back what they heard. Sentences were presented in quiet and the number of correct words were recorded and reported as a percentage (%); higher scores indicate better performance. Speech identification was measured in two conditions: first-ear CI only as well as in the best-aided condition, with the order of testing conditions randomized for each participant. A different sentence list was used for each listening condition. First-ear CI only and best-aided conditions were assessed separately for two reasons. From a clinical standpoint, this separation allows for comparison of hearing performance between these distinct hearing conditions and is therefore standard of care at Swedish Medical Group. This protocol is based on clinical guidelines outlined by Fabry et al. (2009). From a research stand point, the first-ear CI only condition was common to all our participants and limited the variability inherent in the best-aided condition due to multiple device configurations (i.e., CI alone, CI with contralateral hearing aid, or bilateral CIs).

### Cognitive Testing

#### Woodcock-Johnson-IV (WJ-IV)

Of the 18 tests included in the WJ-IV battery, six were selected to specifically evaluate auditory WM, visual memory, spatial relations, perceptual speed, executive processing, attention, concentration, and processing speed (see Table 2 for a complete description of the constructs measured by each test; McGrew et al., 2014). All participants were administered sample items and a practice trial for all six of the cognitive tests prior to recording their actual performance scores. Untimed WJ-IV test items (i.e., perceptual speed tests: Letter Pattern Matching, Number Pattern Matching, Pair Cancellation) are arranged in order of difficulty and therefore had ceiling and basal (i.e., floor) levels. The ceiling level refers to the item at which the participant had no chance of answering correctly (i.e., the highest level at which difficult items were answered incorrectly), while the basal level reflects the items at which the participant had a 100% chance of answering correctly (i.e., the lowest level at which easy items were answered correctly). Tests that had a time requirement of 3 min did not have ceiling and basal levels. Actual performance scores were clustered using the WJ-IV software. Cluster scores represent a combination of individual WJ-IV tests measuring the same cognitive skills, and are used to avoid generalizing the scores from a single narrow ability to a multifaceted skill. Cluster interpretation therefore achieves higher validity (Schrank et al., 2014). Cluster scores were calculated based on the average scores of the individual tests contributing to a particular cognitive skill as determined by the WJ-IV software (McGrew et al., 2014). Cluster scores are summarized in Table 3.

**Table 2:** Description of constructs measured by cognitive test.

Test(s)	Construct(s) measured	Definition
Numbers Reversed Test	Auditory-visual working memory	The ability to temporarily store and manipulate auditory-visual information (i.e., words or numbers).
	Cognitive efficiency	A combination of processing speed and short-term working memory when completing cognitive tasks.
Picture Recognition Test	Visual memory	Retrieval of stored visual stimuli and representations.
Visualization Parts A and B	Spatial relations	(A) Visual feature detection and matching.
		(B) Manipulation (i.e., rotation) of visual stimuli.
Letter Pattern Matching, Number Pattern Matching, Pair Cancellation Task	Perceptual speed	The ability to perform rapid symbol tasks related to orthographic processing.
	Attention	Holding important or relevant stimuli in immediate awareness.
	Concentration	The ability to focus attention on important or relevant stimuli.
Visual Numbers Reversed	Visual working memory	The ability to temporarily store and manipulate visual information (i.e., numbers).


**Table 3:** WJ-IV cluster scores and corresponding aggregate tasks.

Cluster	WJ-IV Tests
**Cognitive processing speed:** demonstrates the ability to quickly perform simple and complex cognitive tasks when controlling sustained attention and concentration.	Letter-Pattern Matching Pair Cancellation
**Visual processing:** demonstrates the ability to perceive, analyze, synthesize and think with visual patterns including the ability to store and recall visual representations.	Visualization Picture Recognition
**Perceptual speed:** demonstrates the ability to perform rapid symbol tasks related to orthographic processing which is important for encoding and decoding information.	Letter-Pattern Matching Number-Pattern Matching
**Cognitive efficiency:** demonstrates cognitive processing speed and short-term working memory. Provides insight into a person’s capacity to hold information in conscious awareness, perform automatic task rapidly and accurately, mental manipulation of information to solve tasks and achieve a goal and controlled attention.	Letter-Pattern Matching Numbers Reversed Number-Pattern Matching


##### Auditory-visual working memory

The WJ-IV Numbers Reversed Test measures short-term WM capabilities (McGrew et al., 2014; Schrank et al., 2014), although it is important to note that a backwards digit span test can be described as a measure of short-term memory rather than WM in normal-hearing young adults (St Clair-Thompson, 2010). However, because of the complexity of auditory-visual functions, this task also inevitably measures cognitive efficiency (see Table 2; Vernon, 1983; McGrew et al., 2014). Participants were asked to repeat a set of numbers that were read aloud (1 s per number) backwards. For example, if the set was “1, 3, 5, 7,” the correct response would have been “7, 5, 3, 1.” This test was conducted in accordance with the WJ-IV’s specifications for HI individuals in that the test was administered verbally by an examiner at a conversational distance, rather than by CD recording (Mather and Wendling, 2014; p. 48), and in a room with minimal visual distractions and no background noise. This recommended protocol modification provided each subject with the possibility of using speech reading abilities to complete this task. Examiners were trained in test presentation with the instructions outlined in the WJ-IV manual. These instructions included allowing 1 s per number and ensuring that their voice was lowered for the last number in each set to indicate the end of the number series. Participants were given one practice trial consisting of a three-digit number set (i.e., 2-4-6). After the practice trial, testing began with number sets of three digits (i.e., 2-4-6) for five consecutive trials before increasing in difficulty to four digits (i.e., 1-3-5-7) for another five consecutive trials and so on, with the most difficult set containing eight digits. Participants received one point per correct trial. Participants continued with the test until the highest five consecutive items were answered incorrectly (i.e., the ceiling level). The basal level for this test was reached when the five lowest-numbered items (i.e., the five test items with the lowest level of difficulty) were answered correctly or until Item 1 was administered. Higher scores indicate better performance.

##### Visual working memory

An in-house visual analog of the WJ-IV Auditory Numbers Reversed Test was created to assess WM capability via the visual modality. Similar to the WJ-IV Auditory Numbers Reversed Test, the visual number series were generated using single digit numbers (i.e., 1-9) and no numbers were repeated within a set. These visual numbers sets were then printed on 8.5 × 11′′ white paper with black, 100-point Calibri (body) font. Participants were asked to repeat a set of numbers that were displayed to them on a card for 1 s per number, backwards. If the set was “3, 1, 6, 9,” the correct response would have been “9, 6, 1, 3.” Participants were given one practice trial consisting of a three-digit number set (i.e., 4-2-9). After the practice trial, testing began with number sets of three digits (i.e., 7-1-6) for five consecutive trials before increasing in difficulty to four digits (i.e., 8-3-1-7) for another five consecutive trials and so on, with the most difficult set containing eight digits. Participants continued with the exam until the highest five consecutive problems were answered incorrectly (i.e., the ceiling level). The basal level for this test was reached when the five lowest-numbered items were answered correctly or until Item 1 was administered. Higher scores indicate better performance. There were no common number series test items to the auditory-visual and visual WM tests. An alternate VWM test using speech reading abilities with no auditory input was considered. However, given that Woodward and Barber (1960) estimated that 60% of speech sounds are not visible with lip-reading, we opted not to include this condition (a visual-only, non-auditory, speech-reading option) for WM as we wanted to ensure that the items to be recalled were accessible to the greatest degree possible. The comparisons, therefore, between visual-only and auditory-visual should be interpreted with the understanding that the visual testing was performed differently between the two tasks.

##### Visual memory

The WJ-IV Picture Recognition test assesses visual memory for pictures of objects (Schrank et al., 2014). After the practice trial, participants were shown a collection of similar objects on one page for 5 s (e.g., a collection of bowls). They were then presented a new page with a mix of objects, some objects from the previous page and others that were not on the previous page (but similar in nature; e.g., the new objects on the page were also bowls). The participant was then asked “Which two/three/four did you see?” This task became increasingly difficult by asking the participant to identify more items throughout the task (i.e., two, then three, then four), while the number of distractor objects increased as well (i.e., identifying the correct items out of a total of 3-7 objects). Participants received one point if they correctly named all objects shown on the first page. Participants continued with the exam until the highest six consecutive problems were answered incorrectly (i.e., the ceiling level) or until all the problems were presented. The basal level for this test was achieved when the lowest six highest-numbered items were answered correctly, or when Item 1 was administered. Higher scores indicate better performance.

##### Spatial relations

The WJ-IV Visualization Parts A (Spatial Relations) and B (Block Rotation) test measures visualization and spatial ability (McGrew et al., 2014; Schrank et al., 2014). In Part A, participants were asked to identify two or three pieces that form a complete target shape. In Part B, participants were asked to perform block rotation by identifying two block patterns that match the target pattern. Part A and B became increasingly difficult by the flipping and rotating of pieces that complete the target shape (i.e., Part A) or pattern (i.e., Part B). For both part A and B, participants received one point per correct response. Both of these parts were administered until the highest five consecutive problems were answered incorrectly (i.e., the ceiling level) or until all of the problems were administered. There was no basal level for this task.

##### Perceptual speed

Three perceptual speed tests were administered: WJ-IV Letter Pattern Matching, WJ-IV Number Pattern Matching and WJ-IV Pair Cancellation Task. Similar to the Numbers Reversed task, the Letter Pattern Matching and Number Pattern Matching tasks measure both processing speed and cognitive efficiency (Mather and Wendling, 2014). All three tests were timed with participants having a time limit of 3 min to complete the task. There were no ceiling or basal levels for these tasks.

##### Letter pattern matching

Letter pattern matching assesses perceptual speed for letter identification. After being administered practice items, participants were asked to identify identical letters or letter groups in a line of six items. For example, participants would circle the “z” in the following: “M z S l z k” or “ack” in: “ack akc cka kca ack kcc.” Participants received one point per correct item (i.e., correct identification of the target letters). There were no ceiling or basal levels for this task.

##### Number pattern matching

Number pattern matching assesses perceptual speed for number identification. Participants were asked to identify identical numbers or number groups in a line of six items. For example, subjects would circle the “6” in the following: “2 6 7 6 3 0” or “385” in: “583 385 358 385 538 835.” This test was administered given that the measures of visual and auditory WM rely on number identification. They received one point per correct item (i.e., correct identification of the target numbers); there were no ceiling or basal levels for this task.

##### Pair cancellation task

The pair cancellation task assesses executive processing, attention, concentration, and processing speed. Participants were asked to identify a specific pattern of two objects in a line of 23 objects. Three types of objects were presented: a ball, a dog, and a cup. The participant was instructed to find and circle the same pattern, “ball, dog,” as quickly as possible in 3 min. If all 90 items were completed in less than 3 min, the total time taken was recorded and included in the calculated score. Participants received one point per correctly identified pair; there were no ceiling or basal levels for this task.

### Questionnaires

The Glasgow Benefit Inventory (GBI) is an 18-item self-assessment scale designed to measure benefit following otorhinolaryngological procedures (Robinson et al., 1996), including cochlear implantation (Ho et al., 2009). There are three subscale scores: general subscale (12 questions), social support (3 questions), and physical health (three questions), all of which address an aspect of how the procedure has benefited the patient. Scores can range from -100 to +100, with higher scores indicating greater benefit of cochlear implantation.

#### The Nijmegen Cochlear Implant Questionnaire (NCIQ)

It is a 60-item self-assessment scale intended for CI users, measuring three health-related quality of life (HRQoL) domains: Physical, Psychological, and Social (Hinderink et al., 2000). Within the Physical domain there are three subdomains: Basic Sound Perception, Advanced Sound Perception, and Speech Production. The Psychological domain measures Self-esteem, and within the Social Domain there are two subdomains: Activity Limitation and Social Interaction. Higher scores indicate better HRQoL.

### Statistical Analyses

Statistical analyses were conducted in SPSS 22.0 (Corporation, 2013). Normality was assessed for all data using the Shapiro–Wilk test. For variables deemed to violate the assumption of normality, non-parametric tests (i.e., Spearman’s Rho) were performed. For all other variables, parametric tests were performed (i.e., repeated measures ANOVA and Pearson correlations). Relationships between AzBio speech perception, cognitive measures, and questionnaire scores were assessed using Pearson’s correlations. Previous research indicates demographic variables such as age, IQ and duration of hearing loss impact CI speech perception. Correlation analyses indicated a significant relationship between age and CI speech perception (AzBio performance: first-ear CI only: *r* = -0.648, *p* = 0.001; best-aided: *r* = -0.536, *p* = 0.012) but not between speech perception and IQ (all *r* ≤ 0.142, all *p* ≥ 0.575) or duration of hearing loss (all *r* ≤ 0.113, all *p* ≥ 0.625). All correlations with AzBio measures, both first-ear CI only and best-aided, were subjected to partial correlations controlling for age. The Physical Health and Social Support subscales of the GBI were not normally distributed and hence subjected to correlational analyses using Spearman’s Rho. All reported statistics reflect two-tailed significance values. Bonferroni corrections were applied when needed.

### Statistical Correction

One unilateral CI subject scored greater than three standard deviations below the mean of best-aided performance. To preserve our sample size for analyses with best-aided performance, a new value was calculated using the mean of the distribution and subtracting two standard deviations. Although scores on the AzBio sentences task are the same for the first-ear CI and best-aided conditions in unilateral CI users (given the absence of a contralateral device), this adjustment was not applied for the first-ear CI condition because the original score was within two standard deviations of the mean of first-ear CI performance. This technique is typically employed for small samples in which there are one to two outliers (Field, 2013).

## Results

**FIGURE 1:**
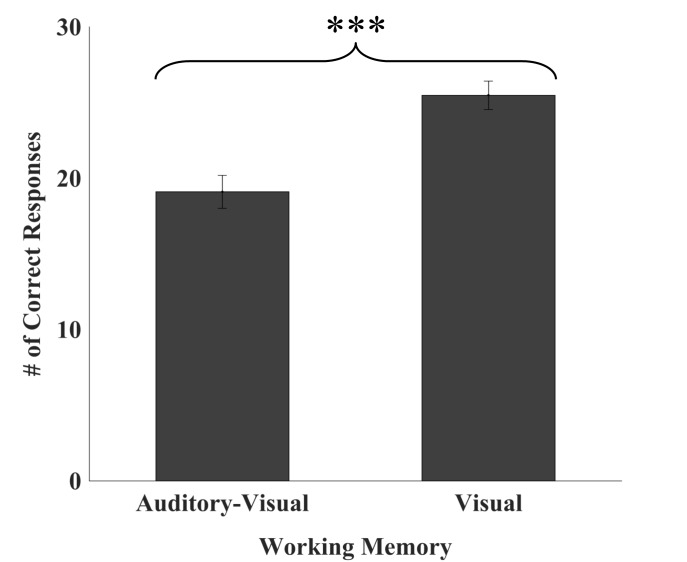
Number of auditory-visual versus visual working memory items recalled. High-performing CI users recalled significantly more visually presented items relative to auditory-visually presented items. Error bars represent 95% confidence intervals. ^∗∗∗^*p* < 0.001.

In high-performing CI users, WM recall was impacted by modality of stimuli presentation, with a greater number of visually presented items correctly recalled than those presented in the auditory-visual modality (*F*_1,20_) = 26.748, *p* < 0.001; see Figure 1. There was a moderate, positive relationship between VWM and AVWM (*r* = 0.427, *p* = 0.05).

### Relationship Between Speech Scores and Cognitive Scores

#### First-Ear CI Only

**FIGURE 2:**
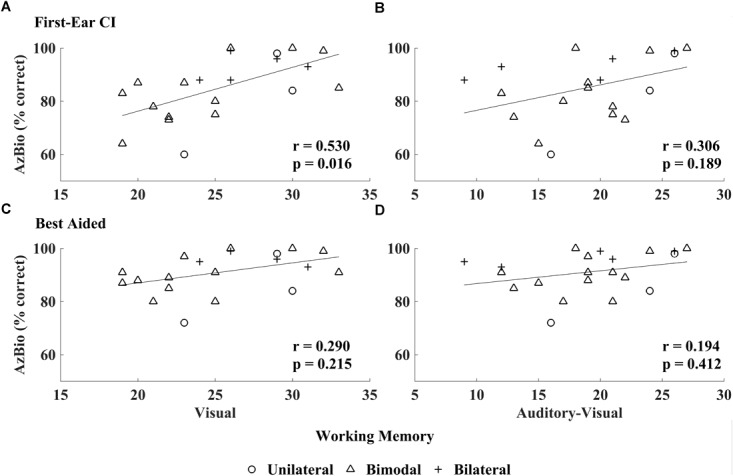
Relationship between CI speech perception in quiet and working memory scores: visual working memory (VWM) and auditory-visual working memory (AVWM). AzBio scores obtained in the first-ear CI test condition related to VWM **(A)** unlike AVWM **(B)**; best-aided speech perception (i.e., unilateral CI, bimodal CI and HA, bilateral CI) related to neither VWM **(C)** nor AVWM **(D)**.

Visual working memory was correlated with speech perception in quiet as measured by the AzBio sentences (*r* = 0.530, *p* = 0.016), while AVWM was not (*r* = 0.306, *p* = 0.189; see Figure 2). No significant association was found between the WJ-IV cluster scores (i.e., cognitive processing speed, visual processing, perceptual speed, or cognitive efficiency), nor IQ and first-ear CI speech perception as measured by AzBio (all *r* ≤0.251, *p* ≥0.314, see Table 4).

**Table 4:** Correlations between AzBio speech scores (controlled for age), non-verbal IQ, and cognitive cluster scores.

	AzBio first-ear CI only	AzBio best aided
Non-verbal IQ	*r* = -0.119 *p* = 0.639	*r* = 0.142 *p* = 0.575
Sound processing (speed)	*r* = 0.171 *p* = 0.499	*r* = 0.182 *p* = 0.471
Visual processing (speed) *N* = 20	*r* = 0.198 *p* = 0.431	*r* = 0.091 *p* = 0.719
Cognitive efficiency^∗^	ρ = 0.165 *p* = 0.474	ρ = 0.144 *p* = 0.533
Perceptual speed	*r* = 0.158 *p* = 0.532	*r* = 0.120 *p* = 0.636


*n = 21 unless otherwise stated. ^∗^Spearman’s rho reported due to violation of normality assumption.*

#### Best-Aided Condition

AzBio speech perception, as measured in the best-aided condition, did not relate with any of the WM measures (VWM: *r* = 0.290, *p* = 0.215; AVWM: *r* = 0.194, *p* = 0.412; see Figure 2), WJ-IV cluster scores (all *r* ≤0.182, *p* ≥0.471) or IQ measures (*r* = 0.142, *p* = 0.575; see Table 4).

### Relationship Between Subjective and Objective Measures of Quality of Life and Hearing Performance

#### First-Ear CI Only

No relationships were observed between first-ear CI only speech perception (i.e., AzBio) and the Glasgow Benefit Inventory (GBI) total score or its subscales, or the Nijmegen Cochlear Implant Questionnaire (NCIQ) subscales (see Table 5).

**Table 5:** Correlations between AzBio speech scores (controlled for age), GBI, and NCIQ questionnaires.

	AzBio first-ear CI only	AzBio best aided
**GBI**		
Total score	*r* = 0.411 *p* = 0.080	***r* = 0.516** ***p* = 0.024^∗^**
General subscale	*r* = 0.354 *p* = 0.137	***r* = 0.560** ***p* = 0.013^∗^**
Social support	ρ = 0.323 *p* = 0.178	ρ = -0.012 *p* = 0.962
Physical health	ρ = 0.199 *p* = 0.414	ρ = -0.199 *p* = 0.414
**NCIQ**		
Basic sound perception	ρ = -0.235 *p* = 0.332	ρ = -0.189 *p* = 0.439
Advanced sound perception	*r* = 0.134 *p* = 0.585	*r* = 0.354 *p* = 0.137
Speech production	*r* = -0.065 *p* = 0.791	*r* = 0.183 *p* = 0.453
Self-esteem	*r* = -0.263 *p* = 0.277	*r* = 0.022 *p* = 0.930
Activity limitation	*r* = 0.284 *p* = 0.239	*r* = 0.453 *p* = 0.051
Social interaction	*r* = 0.057 *p* = 0.818	*r* = 0.200 *p* = 0.411


*^∗^p ≤ 0.05, n = 20. Bold values represent a statistically significant result. Glasgow Benefit Inventory Social Support, Physical Health, and NCIQ Basic Sound Perception subscales were not normally distributed so Spearman’s rho (ρ) is reported. Bonferroni correction was p ≤0.025 for the GBI and p ≤0.01 for the NCIQ.*

#### Best-Aided Condition

Best-aided speech perception assessed with AzBio sentence scores was correlated with both the GBI total score (*r* = 0.516, *p* = 0.024) and the General Subscale of the GBI (*r* = 0.560, *p* = 0.013). No relationships were observed between best-aided speech perception and the Physical Health and Social Support subscales of the GBI, or with the NCIQ subscales (see Table 5).

## Discussion

Here, we demonstrate that high-performing CI users recall more items presented in a visual modality relative to those presented in an auditory-visual modality. Secondly, we show that after controlling for age, VWM relates to speech perception in quiet (i.e., AzBio sentences) for high-performing CI users in the first-ear CI condition, but not in the best-aided condition. These results support the hypothesis that the visual presentation of WM tests may provide a useful measure of cognitive function in CI recipients, and thus may increase the potential for predicting speech perception outcomes. Additionally, we observed no association between CI speech perception ability in quiet and measures of IQ, processing speed or cognitive efficiency. Importantly, processing speed, assessed using visual presentation of materials, unlike VWM, did not relate to speech perception performance in our CI users. Our results suggest that when aiming to predict CI speech perception, presentation modality and cognitive test, rather than presentation modality alone, need to be considered. Lastly, high-performing CI users’ subjective report of benefit (GBI) relates to speech perception for the best-aided condition but not the first-ear CI condition.

### Cognitive Ability in High-Performing CI Users

Cognitive ability is of particular interest in CI recipients given that increased cognitive resources are required for speech perception in HI populations compared to NH individuals (Pichora-Fuller et al., 2016). This is because understanding a complex auditory signal, such as speech, requires the interplay of perceptual and cognitive skills (Daneman and Merikle, 1996; Waters and Caplan, 2005; Wingfield and Tun, 2007; Başkent, 2012). When signals are heard clearly, those signals are used to comprehend language and in turn, communicate (i.e., a bottom–up process). When signals are unclear (i.e., degraded because of hearing loss), linguistic knowledge and environmental context are relied upon to fill in the gaps and deduce the original signal (i.e., a top–down process; Wingfield et al., 2006; Başkent, 2012). For HI individuals, top–down signal deduction (also referred to as phonemic restoration) results in effortful listening as well as a reduction in available cognitive resources. Available cognitive resources are reduced during degraded speech perception tasks, with more resources being reallocated toward achieving proper speech intelligibility, resulting in fewer resources available for other mental tasks such as comprehension and memory of language (Pichora-Fuller, 2006a).

Profoundly deafened individuals may develop compensatory strategies to improve speech perception ability given degraded auditory information during the period of deafness, as well as when interpreting the coarse auditory information provided by a cochlear implant (Lazard et al., 2012a). Growing evidence points to post-lingually deafened CI users having superior multisensory auditory-visual integration ability, underscoring the potential positive benefits of this cross-modal plasticity (Rouger et al., 2007; Strelnikov et al., 2009; Barone and Deguine, 2011; for review see Sharma and Glick, 2016). The complexity of the changes in cortical neural wiring during hearing loss and subsequent auditory restoration may, however, be maladaptive and impede speech perception with a CI (Doucet et al., 2006; Sandmann et al., 2012). As such, assessment of CI users’ cognitive abilities is needed to better understand the contribution of cognition (i.e., WM) to speech perception in this population.

To accurately measure cognition in CI users and reduce the impact of hearing loss and distorted signals on test performance, modality of presentation for cognitive test instruments must be considered. So far, outcomes vary in studies exploring the impact of presentation modality as it relates to speech perception for CI users (Heydebrand et al., 2007; Holden et al., 2013; Moberly et al., 2017a,b, 2018c; Pisoni et al., 2018). Previous conflicting outcomes may arise, at least partially, from the variety of test measures used. For example, CI speech perception measures reported in the literature vary from use of monosyllabic words to full sentences. Additionally, within sentence level materials, there is a wide variety of measures (e.g., CUNY sentences, AzBio sentences, HINT sentences, Presto sentences or Harvard Standard sentences). Variability within cognitive test batteries also exists, with several separate assessments of WM and processing speed employed [e.g., California Verbal Learning Test (CVLT), Reading Span (RSpan), Listening Span (LSpan), Wechsler Adult Intelligence Scale (WAIS), Test of Word Reading Efficiency, and Raven’s Progressive Matrices]. Use of demographic variables as covariates can also impact relationships between cognitive measures and speech perception outcomes. Indeed, Heydebrand et al. (2007) showed that specific subtests of the CVLT, when presented in the auditory-visual modality, related to speech perception, whereas Holden et al. (2013) found that inclusion of age, gender, and ethnicity as covariates eliminated this relationship. More recently, performance on the CVLT-Second Edition (CVLT-II), when presented in a visual-only modality, was found to relate to CI speech perception in quiet (Pisoni et al., 2018). However, visual presentation of cognitive test material does not assure a relationship with CI speech perception. For instance, Moberly et al. (2018c) showed a relationship between CI speech perception in quiet and visual digit span, but not with visual symbol span. With our work, we also show a distinction between visual cognitive tests, with CI speech outcomes relating to VWM, but not with other visually-presented tests of processing speed, cognitive efficiency, or IQ. Our results, in addition to results presented by Moberly et al. (2018c), suggest that visual presentation of number recall either in a forward or backward paradigm may provide the greatest clinical relevance among visually-presented cognitive test items for explaining CI speech perception ability.

Unlike our study, the aforementioned research assessed the relationship between cognitive measures and CI speech perception almost exclusively in the best-aided (i.e., everyday listening) condition. While some studies have shown a relationship between CI speech perception and auditory, auditory-visual or visual WM, others have not (Heydebrand et al., 2007; Holden et al., 2013; Moberly et al., 2017a,b, 2018c; Pisoni et al., 2018). Here, we show that in post-lingually deafened high-performing adult CI users, VWM relates to first-ear implanted, but not best-aided, sentence level speech perception (i.e., AzBio sentences in quiet); and AVWM does not relate to either. The relationship between VWM and first-ear CI performance, but not best-aided listening performance, may be due to inherent group variability. For example, in our study, subjects share a common denominator of having a first-ear CI. When considering the best-aided condition, however, subjects can be stratified into three different groups: unilateral CI users (3), bimodal CI with contralateral HA users (13) and bilateral CI users (5). In this data set, bimodal subjects demonstrated a 7% average increase in speech perception between their first-ear CI only and best-aided (CI + HA) condition, whereas bilateral CI users demonstrated a 4% increase between their first-ear CI only and best-aided (bilateral CI) condition. Unilateral CI subjects demonstrated no change in performance, as their first-ear CI performance and their best-aided performance were identical given the absence of a contralateral device. As such, it may be the case that when these three distinct groups are aggregated, this variability erodes the relationship between performance in the best-aided condition and VWM. Given the limited sample size of each group when stratified by listening condition, future work with additional subjects is needed to assess whether there are performance differences between unilateral, bimodal and bilateral CI users.

### High-Performing CI Users’ Self-Reported Benefit

With respect to subjective benefit, high-performing CI users’ responses to the GBI, but not the NCIQ, relate to objective CI speech perception scores (i.e., best-aided AzBio sentences in quiet). These results suggest that the GBI is better suited to capture this patient population’s subjective report of benefit in a way that meaningfully relates to clinical speech measures. As such, the general subscale domain of the GBI may provide additional insight for clinicians when assessing deficits in speech perception in quiet or changes in performance in this patient population. The absent relationship between NCIQ measures and speech perception in high-performing CI users is consistent with the findings of the NCIQ developers, who also showed no relationship between these measures at a static time point (Henkin et al., 2015). Alternatively, Mosnier et al. (2015) demonstrated that the subjective degree of pre-to-post CI perceived improvement, as measured by the NCIQ, correlated with increased pre-CI to 12-month post-CI speech perception scores, suggesting that this questionnaire may be a more useful metric for gauging pre-to-post implantation performance. Relationships have also been found between specific NCIQ subdomains such as Advanced Speech Perception (Capretta and Moberly, 2016) and Basic Speech Perception (Olze et al., 2012), with speech perception in quiet and noise. Additional relationships among other NCIQ subdomains are infrequently found, or they become non-significant after accounting for multiple comparisons (Moberly et al., 2018b). Moreover, a meta-analysis (McRackan et al., 2018) revealed negligible correlations between the quality of life NCIQ measures with word and sentence recognition in quiet, and negligible-to-medium correlations with sentence recognition in noise. Several factors, such as not accounting for age in the analyses or using best-aided or everyday CI settings, instead of first-CI only settings (Capretta and Moberly, 2016), may have influenced the detection of these relationships.

### Clinical Implications

The role of cognition in speech perception has been increasingly elucidated in hearing aid research, with growing evidence that WM measures can provide additional clinical insight for the selection of rehabilitative programs. Indeed, WM scores may be more sensitive to change and more efficacious in assessment of benefit post hearing aid fitting or auditory rehabilitation program than traditional speech testing materials, which routinely encounter ceiling effects (Pichora-Fuller and Souza, 2003; Pichora-Fuller, 2006a,b; Pichora-Fuller and Singh, 2006). While these studies have focused on milder hearing losses than the population described here, ceiling effects on speech perception materials are also a growing clinical phenomenon for CI recipients (Gifford et al., 2008; Auditory Potential LLC, 2011). The initial goals of early single-channel CIs were modest, including sound awareness or closed-set speech perception. With dramatic technological and engineering developments, more complex sentence level materials with the addition of background noise are routinely evaluated in CI users. Akin to hearing aid research documenting the interaction of cognitive skills with an individual’s ability to benefit from fast or slow compression characteristics (Gatehouse et al., 2006; Lunner and Sundewall-Thorén, 2007; Souza and Sirow, 2014), the accurate assessment of cognitive skills in CI users may shed light on why some patients perform better with one processing strategy over another (i.e., HiResP vs. HiResS: paired vs. sequential electrode stimulation patterns). CI user cognitive skill assessment may also provide clinicians with a means to better select and counsel patients on rehabilitative interventions.

### Limitations and Future Directions

There are limitations to the present study as they relate to the participant pool. First, while we considered CI users adequately experienced with 6 or more months of device use, Herzog et al. (2003) and Lenarz et al. (2012) suggest CI performance in quiet may not plateau until 2 years post-implantation, and therefore, the CI experience of the current participant pool may be limited with respect to performance potential. Another limitation to our study is that a NH group was not included as a control for comparison of performance on AVWM versus VWM tasks to the CI participant group. Given that a NH control group was not evaluated, it remains unclear whether performance differences based on presentation modality are phenomena observed in the general population or only in high-performing CI users. Future work testing NH participants as well as lower-performing CI users (i.e., with AzBio performance in quiet scores < 60%) should be considered to further disentangle this variation in AVWM versus VWM performance. Additionally, inherent potential differences in presentation of AVWM versus VWM tasks should also be considered as possibly influencing participant performance outcome. For instance, because tasks were administered by hand by the experimenter, variations in presentation time may have occurred between verbal and manual presentation of test stimuli. Future work with recorded AV materials should be explored to limit the variability of test item presentation between AVWM and VWM tasks. Lastly, while our sample size is not atypical in the CI literature (Herzog et al., 2003; Olze et al., 2011; Capretta and Moberly, 2016), it is relatively small, which may have limited our ability to detect smaller differences. It is therefore possible that with a larger sample size, relationships between other variables (e.g., speech perception and AVWM) could be elucidated. Future research should not only aim for larger samples, which would allow for more complex modeling of auditory-cognitive interactions, but also to test whether unilateral CI, bilateral CIs, or bimodal CI and HA users present with distinct auditory-cognitive profiles.

## Conclusion

Here we show that visual presentation of Numbers Reversed as a measure of WM provides a useful assessment of cognitive abilities in high-performing CI users. Furthermore, VWM potentially provides a more accurate metric of WM as it relates to clinical speech perception compared to measures of AVWM, cognitive efficiency, IQ, and processing speed. Future work aimed at validating this in a larger group of CI users, including low-performing CI users, is needed.

## Author Contributions

JH, EE, SW, and AP-C contributed to conception and design of the study. JH and CH organized the database. AP-C and JH performed the statistical analysis and wrote the first draft of the manuscript. AP-C, JH, and JA analyzed and interpreted the data. All authors contributed to manuscript revision, read and approved the submitted version.

## Conflict of Interest Statement

The authors declare that the research was conducted in the absence of any commercial or financial relationships that could be construed as a potential conflict of interest.

## References

[B1] Ahissar M. Nahum M. Nelken I. Hochstein S. (2008). Reverse hierarchies and sensory learning. *Philos. Trans. R. Soc. Lond. B Biol. Sci.* 364 285–299. 10.1098/rstb.2008.0253 18986968 PMC2674477

[B2] Akeroyd M. A. (2008). Are individual differences in speech reception related to individual differences in cognitive ability? A survey of twenty experimental studies with normal and hearing-impaired adults. *Int. J. Audiol.* 47 S53–S71. 10.1080/14992020802301142 19012113

[B3] Arlinger S. Lunner T. Lyxell B. Pichora-Fuller M. K. (2009). The emergence of cognitive hearing science. *Scand. J. Psychol.* 50 371–384. 10.1111/j.1467-9450.2009.00753.x 19778385

[B4] Auditory Potential LLC (2011). *Minimum Speech Test Battery (MSTB) for Adult Cochlear Implant Users.* Goodyear, AZ: Auditory Potential LLC.

[B5] Barone P. Deguine O. (2011). “Multisensory processing in cochlear implant listeners,” in *Auditory Prostheses: Cochlear Implants and Beyond*, eds Zeng F. G. Popper A. N. Fay R. (New York, NY: Springer Handbook of Auditory Research), 365–382.

[B6] Başkent D. (2012). Effect of speech degradation on top-down repair: phonemic restoration with simulations of cochlear implants and combined electric–acoustic stimulation. *J. Assoc. Res. Otolaryngol.* 13 683–692. 10.1007/s10162-012-0334-3 22569838 PMC3441953

[B7] Blamey P. Arndt P. Bergeron F. Bredberg G. Brimacombe J. Facer G. (1996). Factors affecting auditory performance of postlinguistically deaf adults using cochlear implants. *Audiol. Neurotol.* 1 293–306. 10.1159/000259212 9390810

[B8] Brown L. Sherbenou R. J. Johnsen S. K. (2010). *TONI-4 Test of Nonverbal Intelligence.* Austin, TX: Pro-Ed.

[B9] Capretta N. R. Moberly A. C. (2016). Does quality of life depend on speech recognition performance for adult cochlear implant users? *Laryngoscope* 126 699–706. 10.1002/lary.25525 26256441

[B10] Collison E. A. Munson B. Carney A. E. (2004). Relations among linguistic and cognitive skills and spoken word recognition in adults with cochlear implants. *J. Speech Lang. Hear. Res.* 47 496–508. 10.1044/1092-4388(2004/039) 15212564

[B11] Corporation I. (2013). *IBM SPSS Statistics for Windows, Version 22.0.* Armonk, NY: IBM Corp.

[B12] Daneman M. Merikle P. M. (1996). Working memory and language comprehension: a meta-analysis. *Psychon. Bull. Rev.* 3 422–433. 10.3758/BF03214546 24213976

[B13] Dawson P. W. Mckay C. M. Busby P. A. Clark G. M. (2002). Rate-of-processing ability in children using cochlear implants and its relevance to speech perception. *Cochlear Implants Int.* 3 126–138. 10.1179/cim.2002.3.2.126 18792119

[B14] Doucet M. Bergeron F. Lassonde M. Ferron P. Lepore F. (2006). Cross-modal reorganization and speech perception in cochlear implant users. *Brain* 129 3376–3383. 10.1093/brain/awl264 17003067

[B15] Dowell R. C. (2012). “Evidence about the effectiveness of cochlear implants for adults,” in *Evidence-Based Practice in Audiology*, eds Wong L. Hickson L. (San Diego, CA: Plural Publishing), 141–165.

[B16] Dupuis K. Pichora-Fuller M. K. Chasteen A. L. Marchuk V. Singh G. Smith S. L. (2015). Effects of hearing and vision impairments on the montreal cognitive assessment. *Aging Neuropsychol. Cogn.* 22 413–437. 10.1080/13825585.2014.968084 25325767

[B17] Fabry D. Firszt J. B. Gifford R. H. Holden L. K. Koch D. (2009). Evaluating speech perception benefit in adult cochlear implant recipients. *Audiol. Today* 21 36–43.

[B18] Field A. (2013). *Discovering Statistics Using IBM SPSS Statistics.* London: Sage.

[B19] Füllgrabe C. Rosen S. (2016). “Investigating the role of working memory in speech-in-noise identification for listeners with normal hearing,” in *Physiology, Psychoacoustics and Cognition in Normal and Impaired Hearing* Vol. 894 eds van Dijk P. Başkent D. Gaudrain E. de Kleine E. Wagner A. Lanting C. (Cham: Springer), 29–36. 10.1007/978-3-319-25474-6_4 PMC5714061 27080643

[B20] Gajadeera E. A. Galvin K. L. Dowell R. C. Busby P. A. (2017). The change in electrical stimulation levels during 24 months postimplantation for a large cohort of adults using the Nucleus^®^ cochlear implant. *Ear Hear.* 38 357–367. 10.1097/AUD.0000000000000405 28166089

[B21] Gantz B. J. Woodworth G. G. Knutson J. F. Abbas P. J. Tyler R. S. (1993). Multivariate predictors of audiological success with multichannel cochlear implants. *Ann. Otol. Rhinol. Laryngol.* 102 909–916. 10.1177/000348949310201201 8285510

[B22] Gatehouse S. Naylor G. Elberling C. (2006). Linear and nonlinear hearing aid fittings–1. Patterns of benefit: adaptación de auxiliares auditivos lineales y no lineales–1. Patrones de beneficio. *Int. J. Audiol.* 45 130–152. 10.1080/14992020500429518 16579490

[B23] Gifford R. H. Shallop J. K. Peterson A. M. (2008). Speech recognition materials and ceiling effects: considerations for cochlear implant programs. *Audiol. Neurotol.* 13 193–205. 10.1159/000113510 18212519

[B24] Henkin Y. Yaar-Soffer Y. Steinberg M. Muchnik C. (2015). Neural correlates of auditory-cognitive processing in older adult cochlear implant recipients. *Audiol. Neurotol.* 19 21–26. 10.1159/000371602 25733362

[B25] Herzog M. Schön F. Müller J. Knaus C. Scholtz L. Helms J. (2003). Long term results after cochlear implantation in elderly patients. *Laryngorhinootologie* 82 490–493. 12886496 10.1055/s-2003-40896

[B26] Heydebrand G. Hale S. Potts L. Gotter B. Skinner M. (2007). Cognitive predictors of improvements in adults’ spoken word recognition six months after cochlear implant activation. *Audiol. Neurotol.* 12 254–264. 10.1159/000101473 17406104

[B27] Hinderink J. B. Krabbe P. F. Van Den Broek P. (2000). Development and application of a health-related quality-of-life instrument for adults with cochlear implants: the Nijmegen cochlear implant questionnaire. *Otolaryngol. Head Neck Surg.* 123 756–765. 10.1067/mhn.2000.108203 11112975

[B28] Ho E. C. Monksfield P. Egan E. Reid A. Proops D. (2009). Bilateral bone-anchored hearing aid: impact on quality of life measured with the Glasgow benefit inventory. *Otol. Neurotol.* 30 891–896. 10.1097/MAO.0b013e3181b4ec6f 19692937

[B29] Holden L. K. Finley C. C. Firszt J. B. Holden T. A. Brenner C. Potts L. G. (2013). Factors affecting open-set word recognition in adults with cochlear implants. *Ear Hear.* 34 342–360. 10.1097/AUD.0b013e3182741aa7 23348845 PMC3636188

[B30] Houtgast T. Festen J. M. (2008). On the auditory and cognitive functions that may explain an individual’s elevation of the speech reception threshold in noise. *Int. J. Audiol.* 47 287–295. 10.1080/14992020802127109 18569101

[B31] Hua H. Johansson B. Magnusson L. Lyxell B. Ellis R. J. (2017). Speech recognition and cognitive skills in bimodal cochlear implant users. *J. Speech Lang. Hear. Res.* 60 2752–2763. 10.1044/2017_JSLHR-H-16-0276 28885638

[B32] Humes L. E. Kidd G. R. Lentz J. J. (2013). Auditory and cognitive factors underlying individual differences in aided speech-understanding among older adults. *Front. Syst. Neurosci.* 7:55 10.3389/fnsys.2013.00055 PMC3787592 24098273

[B33] Lazard D. S. Giraud A.-L. Gnansia D. Meyer B. Sterkers O. (2012a). Understanding the deafened brain: implications for cochlear implant rehabilitation. *Eur. Ann. Otorhinolaryngol. Head Neck Dis.* 129 98–103. 10.1016/j.anorl.2011.06.001 22104578

[B34] Lazard D. S. Vincent C. Venail F. Van De Heyning P. Truy E. Sterkers O. (2012b). Pre-, per-and postoperative factors affecting performance of postlinguistically deaf adults using cochlear implants: a new conceptual model over time. *PLoS One* 7:e48739. 10.1371/journal.pone.0048739 23152797 PMC3494723

[B35] Lenarz M. Sönmez H. Joseph G. Büchner A. Lenarz T. (2012). Cochlear implant performance in geriatric patients. *Laryngoscope* 122 1361–1365. 10.1002/lary.23232 22539093

[B36] Lunner T. Sundewall-Thorén E. (2007). Interactions between cognition, compression, and listening conditions: effects on speech-in-noise performance in a two-channel hearing aid. *J. Am. Acad. Audiol.* 18 604–617. 10.3766/jaaa.18.7.7 18236647

[B37] Lyxell B. Andersson J. Andersson U. Arlinger S. Bredberg G. Harder H. (1998). Phonological representation and speech understanding with cochlear implants in deafened adults. *Scand. J. Psychol.* 39 175–179. 10.1111/1467-9450.393075 9800533

[B38] Mather N. Wendling B. J. (2014). *Woodcock Johnson IV Tests of Cognitive Abilities Examiner’s Manual.* Rolling Meadows, IL: Riverside Publishing.

[B39] McGrew K. S. Laforte E. M. Schrank F. A. (2014). *Woodcock Johnson IV Technical Manual.* Rolling Meadows, IL: Riverside Publishing.

[B40] McRackan T. R. Bauschard M. Hatch J. L. Franko-Tobin E. Droghini H. R. Nguyen S. A. (2018). Meta-analysis of quality-of-life improvement after cochlear implantation and associations with speech recognition abilities. *Laryngoscope* 128 982–990. 10.1002/lary.26738 28731538 PMC5776066

[B41] Moberly A. C. Castellanos I. Vasil K. J. Adunka O. F. Pisoni D. B. (2018a). ”Product” versus ”Process” measures in assessing speech recognition outcomes in adults with cochlear implants. *Otol. Neurotol.* 39 e195–e202. 10.1097/MAO.0000000000001694 29342056 PMC5807136

[B42] Moberly A. C. Harris M. S. Boyce L. Vasil K. Wucinich T. Pisoni D. B. (2018b). Relating quality of life to outcomes and predictors in adult cochlear implant users: are we measuring the right things? *Laryngoscope* 128 959–966. 10.1002/lary.26791 28776711 PMC6192249

[B43] Moberly A. C. Pisoni D. B. Harris M. S. (2018c). Visual working memory span in adults with cochlear implants: some preliminary findings. *World J. Otorhinolaryngol. Head Neck Surg.* 3 224–230. 10.1016/j.wjorl.2017.12.003 29780967 PMC5956138

[B44] Moberly A. C. Harris M. S. Boyce L. Nittrouer S. (2017a). Speech recognition in adults with cochlear implants: the effects of working memory, phonological sensitivity, and aging. *J. Speech Lang. Hear. Res.* 60 1046–1061. 10.1044/2016_JSLHR-H-16-0119 28384805 PMC5548076

[B45] Moberly A. C. Houston D. M. Harris M. S. Adunka O. F. Castellanos I. (2017b). Verbal working memory and inhibition-concentration in adults with cochlear implants. *Laryngoscope Investig. Otolaryngol.* 2 254–261. 10.1002/lio2.90 29094068 PMC5655567

[B46] Mosnier I. Bebear J.-P. Marx M. Fraysse B. Truy E. Lina-Granade G. (2015). Improvement of cognitive function after cochlear implantation in elderly patients. *JAMA Otolaryngol. Head Neck Surg.* 141 442–450. 10.1001/jamaoto.2015.129 25763680

[B47] Olze H. Gräbel S. Förster U. Zirke N. Huhnd L. E. Haupt H. (2012). Elderly patients benefit from cochlear implantation regarding auditory rehabilitation, quality of life, tinnitus, and stress. *Laryngoscope* 122 196–203. 10.1002/lary.22356 21997855

[B48] Olze H. Szczepek A. J. Haupt H. Förster U. Zirke N. Gräbel S. (2011). Cochlear implantation has a positive influence on quality of life, tinnitus, and psychological comorbidity. *Laryngoscope* 121 2220–2227. 10.1002/lary.22145 21898434

[B49] Pichora-Fuller M. K. (2006a). “Audition and cognition: what audiologists need to know about listening,” in *Hearing Care for Adults*, eds Palmer C. Seewald R. (Stäfa: Phonak), 71–85.

[B50] Pichora-Fuller M. K. (2006b). Perceptual effort and apparent cognitive decline: implications for audiologic rehabilitation. *Semin. Hear.* 27 284–293. 10.1055/s-2006-954855

[B51] Pichora-Fuller M. K. Kramer S. E. Eckert M. A. Edwards B. Hornsby B. W. Humes L. E. (2016). Hearing impairment and cognitive energy: the framework for understanding effortful listening (FUEL). *Ear Hear.* 37 5S–27S. 10.1097/AUD.0000000000000312 27355771

[B52] Pichora-Fuller M. K. Singh G. (2006). Effects of age on auditory and cognitive processing: implications for hearing aid fitting and audiologic rehabilitation. *Trends Amplif.* 10 29–59. 10.1177/108471380601000103 16528429 PMC4111543

[B53] Pichora-Fuller M. K. Souza P. E. (2003). Effects of aging on auditory processing of speech. *Int. J. Audiol.* 42(Suppl. 2), S11–S16. 10.3109/14992020309074638 12918623

[B54] Pisoni D. B. Broadstock A. Wucinich T. Safdar N. Miller K. Hernandez L. R. (2018). Verbal learning and memory after cochlear implantation in postlingually deaf adults: some new findings with the CVLT-II. *Ear Hear.* 39 720–745. 10.1097/AUD.0000000000000530 29271831 PMC6013309

[B55] Pisoni D. B. Cleary M. (2003). Measures of working memory span and verbal rehearsal speed in deaf children after cochlear implantation. *Ear Hear.* 24 106S–120S. 10.1097/01.AUD.0000051692.05140.8E 12612485 PMC3434463

[B56] Pisoni D. D. Geers A. E. (2000). Working memory in deaf children with cochlear implants: correlations between digit span and measures of spoken language processing. *Ann. Otol. Rhinol. Laryngol. Suppl.* 185 92–93. 10.1177/0003489400109S1240 11141023 PMC3429114

[B57] Punch J. L. Robbins A. M. Myres W. A. Pope M. L. Miyamoto R. T. (1987). Relationships among selected measures of single-channel cochlear implant performance. *Ear Hear.* 8 37–43. 10.1097/00003446-198702000-00007 3556809

[B58] Purdy S. C. Welch D. Giles E. Morgan C. L. A. Tenhagen R. Kuruvilla-Mathew A. (2017). Impact of cognition and noise reduction on speech perception in adults with unilateral cochlear implants. *Cochlear Implants Int.* 18 162–170. 10.1080/14670100.2017.1299393 28335695

[B59] Robinson K. Gatehouse S. Browning G. G. (1996). Measuring patient benefit from otorhinolaryngological surgery and therapy. *Ann. Otol. Rhinol. Laryngol.* 105 415–422. 10.1177/000348949610500601 8638891

[B60] Rouger J. Lagleyre S. Fraysse B. Deneve S. Deguine O. Barone P. (2007). Evidence that cochlear-implanted deaf patients are better multisensory integrators. *Proc. Natl. Acad. Sci. U.S.A.* 104 7295–7300. 10.1073/pnas.0609419104 17404220 PMC1855404

[B61] Sandmann P. Dillier N. Eichele T. Meyer M. Kegel A. Pascual-Marqui R. D. (2012). Visual activation of auditory cortex reflects maladaptive plasticity in cochlear implant users. *Brain* 135(Pt 2), 555–568. 10.1093/brain/awr329 22232592

[B62] Schrank F. A. Mcgrew K. S. Mather N. (2014). *Woodcock-Johnson IV Tests of Cognitive Abilities.* Rolling Meadows, IL: Riverside Publishing.

[B63] Sharma A. Glick H. (2016). Cross-modal re-organization in clinical populations with hearing loss. *Brain Sci.* 6:E4. 10.3390/brainsci6010004 26821049 PMC4810174

[B64] Smith S. L. Pichora-Fuller M. K. (2015). Associations between speech understanding and auditory and visual tests of verbal working memory: effects of linguistic complexity, task, age, and hearing loss. *Front. Psychol.* 6:1394. 10.3389/fpsyg.2015.01394 26441769 PMC4584991

[B65] Souza P. E. Sirow L. (2014). Relating working memory to compression parameters in clinically fit hearing aids. *Am. J. Audiol.* 23 394–401. 10.1044/2014_AJA-14-0006 25123548 PMC4332516

[B66] Spahr A. J. Dorman M. F. Litvak L. M. Van Wie S. Gifford R. H. Loizou P. C. (2012). Development and validation of the AzBio sentence lists. *Ear Hear.* 33 112–117. 10.1097/AUD.0b013e31822c2549 21829134 PMC4643855

[B67] St Clair-Thompson H. L. (2010). Backwards digit recall: a measure of short-term memory or working memory? *Eur. J. Cogn. Psychol.* 22 286–296. 10.1080/09541440902771299

[B68] Stewart R. Wingfield A. (2009). Hearing loss and cognitive effort in older adults’ report accuracy for verbal materials. *J. Am. Acad. Audiol.* 20 147–154. 10.3766/jaaa.20.2.7 19927677 PMC2867098

[B69] Strelnikov K. Rouger J. Demonet J.-F. Lagleyre S. Fraysse B. Deguine O. (2009). Does brain activity at rest reflect adaptive strategies? Evidence from speech processing after cochlear implantation. *Cereb. Cortex* 20 1217–1222. 10.1093/cercor/bhp183 19805418

[B70] van Dijk J. E. Van Olphen A. F. Langereis M. C. Mens L. H. Brokx J. P. Smoorenburg G. F. (1999). Predictors of cochlear implant performance. *Audiology* 38 109–116. 10.3109/00206099909073010 10206520

[B71] van Eijl R. H. Buitenhuis P. J. Stegeman I. Klis S. F. Grolman W. (2016). Systematic review of compound action potentials as predictors for cochlear implant performance. *Laryngoscope* 127 476–487. 10.1002/lary.26154 27804133

[B72] van Rooij J. Plomp R. (1990). Auditive and cognitive factors in speech perception by elderly listeners. II: multivariate analyses. *J. Acoust. Soc. Am.* 88 2611–2624. 10.1121/1.399981 2283434

[B73] Vernon P. A. (1983). Speed of information processing and general intelligence. *Intelligence* 7 53–70. 10.1016/0160-2896(83)90006-5

[B74] Waters G. S. Caplan D. (2005). The relationship between age, processing speed, working memory capacity and language comprehension. *Memory* 13 403–414. 10.1080/09658210344000459 15952262

[B75] Weinstein B. Amsel L. (1986). The relationship between dementia and hearing impairment in the institutionalized elderly. *Cin. Gerontol.* 4 3–15.

[B76] Wingfield A. Tun P. A. (2007). Cognitive supports and cognitive constraints on comprehension of spoken language. *J. Am. Acad. Audiol.* 18 548–558. 10.3766/jaaa.18.7.3 18236643

[B77] Wingfield A. McCoy S. L. Peelle J. E. Tun P. A. Cox C. L. (2006). Effects of adult aging and hearing loss on comprehension of rapid speech varying in syntactic complexity. *J. Am. Acad. Audiol.* 17 487–497. 10.3766/jaaa.17.7.4 16927513

[B78] Woodward M. F. Barber C. G. (1960). Phoneme perception in lipreading. *J. Speech Lang. Hear. Res.* 3 212–222. 10.1044/jshr.0303.212 13845910

[B79] Zekveld A. A. Kramer S. E. Festen J. M. (2011). Cognitive load during speech perception in noise: the influence of age, hearing loss, and cognition on the pupil response. *Ear Hear.* 32 498–510. 10.1097/AUD.0b013e31820512bb 21233711

